# Ethnicity and the first diagnosis of a wide range of cardiovascular diseases: Associations in a linked electronic health record cohort of 1 million patients

**DOI:** 10.1371/journal.pone.0178945

**Published:** 2017-06-09

**Authors:** Julie George, Rohini Mathur, Anoop Dinesh Shah, Mar Pujades-Rodriguez, Spiros Denaxas, Liam Smeeth, Adam Timmis, Harry Hemingway

**Affiliations:** 1 The Farr Institute of Health Informatics Research and the National Institute for Health Research, Biomedical Research Centre, University College London, London, United Kingdom; 2 Electronic Health Records Group, Non-Communicable Disease Epidemiology, London School of Hygiene and Tropical Medicine, London, United Kingdom; 3 Leeds Institute of Biomedical and Clinical Science, University of Leeds, Leeds, United Kingdom; 4 NIHR Cardiovascular Biomedical Research Unit, Barts Heart Centre, London, United Kingdom; Indiana University School of Medicine, UNITED STATES

## Abstract

**Background:**

While the association of ethnic group with individual cardiovascular diseases has been studied, little is known about ethnic differences in the initial lifetime presentation of clinical cardiovascular disease in contemporary populations.

**Methods and results:**

We studied 1,068,318 people, aged ≥30 years and free from diagnosed CVD at baseline (90.9% White, 3.6% South Asian and 2.9% Black), using English linked electronic health records covering primary care, hospital admissions, acute coronary syndrome registry and mortality registry (CALIBER platform). During 5.7 years median follow-up between 1997–2010, 95,224 people experienced an incident cardiovascular diagnosis. 69.9% (67.2%-72.4%) of initial presentation in South Asian <60 yrs were coronary heart disease presentations compared to 47.8% (47.3%-48.3%) in White and 40.1% (36.3%-43.9%) in Black patients. Compared to White patients, Black patients had significantly lower age-sex adjusted hazard ratios (HRs) for initial lifetime presentation of all the coronary disease diagnoses (stable angina HR 0.80 (95% CI 0.68–0.93); unstable angina– 0.75 (0.59–0.97); myocardial infarction 0.49 (0.40–0.62)) while South Asian patients had significantly higher HRs (stable angina– 1.67 (1.52–1.84); unstable angina 1.82 (1.56–2.13); myocardial infarction– 1.67 (1.49–1.87). We found no ethnic differences in initial presentation with heart failure (Black 0.97 (0.79–1.20); S Asian 1.04(0.87–1.26)). Compared to White patients, Black patients were more likely to present with ischaemic stroke (1.24 (0.97–1.58)) and intracerebral haemorrhage (1.44 (0.97–2.12)). Presentation with peripheral arterial disease was less likely for Black (0.63 (0.50–0.80)) and South Asian patients (0.70 (0.57–0.86)) compared with White patients.

**Discussion:**

While we found the anticipated substantial predominance of coronary heart disease presentations in South Asian and predominance of stroke presentations in Black patients, we found no ethnic differences in presentation with heart failure. We consider the public health and research implications of our findings.

**Trial Registration:**

NCT02176174, www.clinicaltrials.gov

## Introduction

Cardiovascular disease accounts for more than a quarter of all deaths in England and Wales[1] and contributes 15% of all disability adjusted life years lost in England.[2]. However the burden of cardiovascular disease (CVD) in the United Kingdom (UK) varies between ethnic groups and type of CVD diagnosis.[3] Compared to White UK residents, South Asian residents have been found to be at increased risk of angina,[4,5] myocardial infarction,[6] coronary heart disease[7,8] but lower risk of heart failure,[9] and out-of-hospital cardiac arrest[10,11]. In contrast, Black residents have been found to be at increased risk of stroke in most but not all studies[7,12,13] and similar or lower risk of heart failure[9,14] and other heart diseases compared to White UK residents.[15,7,11] Studies are lacking on ethnic differences in incidence of some cardiovascular disease diagnoses, specifically peripheral arterial disease, stroke subtypes, and abdominal aortic aneurysm.

Despite the range of studies investigating ethnic differences in incident cardiovascular diseases, most previous studies have looked at individual or a limited number of cardiovascular diseases in isolation.[16–24] It is unknown how UK ethnic groups differ in the cardiovascular disease with which they are first diagnosed across a range of specific acute and chronic disease diagnoses. First lifetime cardiovascular disease diagnosis is a turning point in a patient’s experience, marking the end of the possibility of primary prevention and the beginning of the need to consider secondary prevention, so understanding how these might differ by ethnic group is important. However, comparisons across such a broad range of outcomes need the large sample sizes to reliably identify ethnic differences in association.

We have created a large UK-based prospective cohort using linked electronic health records with detailed information on ethnic group, cardiovascular risk factors and diagnoses in order to address this gap in knowledge.[25] Specifically, we sought to address the following objectives:

1. To examine the proportion of the total burden of CVD comprised by specific cardiovascular diagnoses for White, South Asian and Black patients, the largest ethnic groups in the UK.
2. To determine how South Asian and Black patients differ from White patients in the initial lifetime diagnosis of cardiovascular disease, across a broad range of cardiovascular diagnoses.
3. To determine whether any associations found are independent of common cardiovascular risk factors such as age, sex, social deprivation, hypertension and diabetes.

## Methods

### Data sources

Anonymised patient records were selected from the Clinical disease research using LInked Bespoke studies and Electronic health Records (CALIBER) programme described[25] and validated[26–31] elsewhere. In brief, patients for the cohort were drawn from the Clinical Practice Research Database (CPRD) which also provided data from the primary care medical record. Patients registered in practices submitting linkable data to CPRD, covering approximately 4% of the English population, have been found to be representative of the English population in terms of age, gender and ethnicity.[32,33]

Further data on the cohort patients was drawn from three other linked clinical datasets: the Myocardial Ischaemia National Audit Project (MINAP) registry, Hospital Episodes Statistics (HES) and the UK national death registry from the Office for National Statistics (ONS).

### Study population

We studied 1,068,318 patients registered between January 1997 and March 2010 from 225 general practices across England submitting data to CPRD. We required that at study entry patients were aged ≥30 years, free of diagnosed CVD and had been followed-up for at least one year. We did not impose an upper age limit and the older patient in the cohort was 109. We used the entire medical history available on each patient to confirm they were free of diagnosed CVD. The period covered by medical history prior to study entry ranged from 20 years to the stipulated minimum period of 1 year, which previous research has found to be a sufficient period to ensure accurate assessment of baseline history of prior diagnoses.[34] Women who were pregnant in the 6 months before study entry were excluded, as were patients with no ethnicity recorded. (See S1 Fig for study flow diagram.) We used an open cohort design, so patients entered the study when they met the inclusion criteria. Patients were censored on the earliest date from among: the date of first CVD diagnosis, date of death from other causes, date leaving the practice or date of last practice data collection.

### Exposure variable–ethnic group

In the United Kingdom, recording of patients’ ethnic group has been mandated in the National Health Service since 1991. Patients are asked to self-classify, specifying the ethnic group to which they belong when they access either primary or secondary care.

The completeness of ethnicity data has increased over time, with substantial increases in HES since 2000[35] and in CPRD since 2006 when recording in primary care was incentivised.[33] We used information on ethnic group recorded in both CPRD (47%) and HES (53%), resolving any conflicts between the two data sources using a defined and previously validated algorithm, which found distribution of ethnic groups similar to the national UK Census.[35] (See S2 Fig for algorithm and source of ethnicity codes.) Patients were categorised as White, South Asian, Black, or Other/Mixed ethnic groups; these groups reflect the most prevalent ethnic groups in the 2011 Census in England and Wales.[36] The White group included White British, White European and other White Groups. South Asian included patients from Indian, Pakistani, Bangladeshi and other Asian ethnic groups, including Asian British. Black included those belonging to African, Caribbean or other Black groups, including Black British. The Other/ Mixed group included those from any mixed ethnic group and other small minority ethnic groups, including Japanese and Chinese.

### Covariates

Baseline cardiovascular risk factors were obtained from CPRD, recorded during primary care consultations. For body mass index (BMI), systolic blood pressure (SBP), total cholesterol (TChol) and HDL cholesterol (HDL), the most recent measurement recorded up to one year before study entry was used as the baseline value. Patients were identified as diabetic if there was a diagnosis of diabetes at any point in the prior medical record, as defined previously by Shah et al.[37] Similarly, smoking status was determined using the entire prior record to classify patients as never-smokers, ex-smokers or current smokers at baseline. Deprivation, divided into quintiles, was measured using the Index of Multiple Deprivation (IMD)[38], a neighbourhood deprivation score combining indices of unemployment, crime, income, education and other markers of social inequality.

Our treatment variables, also obtained from CPRD, [39] included receipt of a repeat prescription (defined as two or more prescriptions in the year prior to study entry) of statins or blood pressure lowering medication (thiazide diuretics, beta-blockers, angiotensin converting enzyme-inhibitors, angiotensin receptor blockers, or calcium-channel blockers) at baseline. We additionally included use of oral contraceptives or hormone replacement therapy (HRT) in women. Variable definitions can be found at http://www.caliberresearch.org/portal/.

### Outcomes

As described in previous papers,[27,30,31] our primary endpoints were defined as the first recorded diagnosis of the 12 most common symptomatic manifestations of CVD, irrespective of underlying disease mechanism, arising from pathology in the head, heart, abdomen or legs. The first diagnosis could occur in primary care, secondary care or at death. We included the following CVDs: stable angina, unstable angina, non-fatal myocardial infarction (MI), unheralded coronary death (UCD), heart failure, a composite of cardiac arrest, ventricular arrhythmia and sudden cardiac death (SCD), transient ischaemic attack (TIA), ischaemic stroke, subarachnoid haemorrhage (SAH), intracerebral haemorrhage, abdominal aortic aneurysm (AAA), peripheral arterial disease (PAD), and other deaths. We combined stroke not otherwise specified with ischaemic stroke, as previous research has identified the large majority of strokes are ischaemic.[40] Coronary heart disease not otherwise specified (CHD NOS) was also studied but kept separate from other coronary diagnoses in the analysis. We classified events as fatal where a death record exists for the same calendar date. Overview of codes and data sources used to define cardiovascular endpoints has been published previously.[30]

### Statistical analysis

Descriptive statistics were used to compare baseline demographic characteristics, risk factors and the number of primary care consultations, and prescribed medication in the year prior to entry by ethnic group. We also analysed the proportion of total CVD that individual cardiovascular diagnoses comprised for each ethnic group, within three broad age bands. Continuous variables are presented as mean while categorical variables are presented as percentages; 95% confidence intervals are given for all descriptive variables and hazard ratios, unless otherwise stated.

In primary analyses, we investigated the association of ethnic group with each CVD outcome across all patients, using *White* as the reference group. Hazard ratios (HRs) were based on disease-specific Cox models with length of follow-up as the timescale, adjusted for age (linear and quadratic term), sex, and stratified by primary care practice. The association of ethnic group with the range of endpoints was analysed in a competing risk framework, i.e. only one of the range of diseases can be the initial diagnosis of cardiovascular disease, with other diagnoses competing to be that first diagnosis. Associations for the mixed/other ethnic group were estimated but are not presented.

In secondary analyses, we investigated the association of ethnic group after adjustment for classical CVD risk-factors (deprivation, smoking status, SBP, diabetes, BMI, total cholesterol, HDL cholesterol), and baseline treatment with blood-pressure lowering medication, statins, and female hormones. We also examined modification of these associations by baseline age group and gender.

Missing values in covariates were handled using multiple imputation for all analyses. (See S1 Appendix for details on our approach to imputation.) The proportional hazards assumption was tested by plotting the Schoenfield residuals for all endpoints, comparing South Asian and Black patients to White. The assumption was met for all endpoints for all ethnic groups.

### Sensitivity analyses

In sensitivity analyses, associations were examined in complete cases and in analyses where we restricted endpoints to those recorded in secondary care and mortality data or mortality data alone. From 1^st^ April 2004, primary care practices began receiving substantial financial rewards for performance in chronic disease management; [41] from 1^st^ April 2006 they further received rewards for recording ethnic group. We therefore also compared associations between ethnic group and initial CVD diagnoses before and after 1^st^ April 2006.

All the analyses were performed with Stata 12 or R 3.0.

### Ethics

The study was approved by the Independent Scientific Advisory Committee (ISAC) of the Medicines and Healthcare Products Regulatory Agency (protocol 12_117) and the MINAP Academic Group. The study was registered at clinicaltrials.gov (trial registration NCT02176174).

## Results

The study cohort comprised 971,283 White, 38,292 South Asian, 30,896 Black and 27,847 Mixed/Other patients, with 6,023,720, 155,278, 133,577 and 114,735 person years of observation respectively. The ethnic group distribution of the cohort was broadly similar to the English population of the same age over the period of the cohort.[36,42] A total of 95,224 cardiovascular events were recorded, 96.2% in White, 1.9% in South Asian, 0.9% in Black and 1.1% in Mixed/Other patients. All further results are reported for the White, South Asian and Black patients only.

White patients were followed up for a median of 6.1 years, while follow-up time was notably shorter for South Asian (2.6 years), and Black patients (3.1 years). (Table 1; see S1 Table for baseline data by age within ethnic group.) Compared with White patients, South Asian and Black patients were significantly younger at study entry, and were more likely to live in the most deprived areas. They were also more likely to be never smokers and to have diabetes and a statin prescription. South Asian patients were less likely to be hypertensive or have BP-lowering medication prescribed, while Black patients were somewhat more likely to be hypertensive, had lower baseline SBP and were prescribed BP medication in similar proportion to White patients. BMI was broadly similar in all three groups.

**Table 1:** Baseline patient characteristics by ethnic group.

	White	South Asian	Black	Other	Total
	N = 971,283 (90.9%)	N = 38,292 (3.6%)	N = 30,896 (2.9%)	N = 27,847 (2.6%)	N = 1,068,318
Observation time in years, median (IQR)	6.1 (2.1–10.2)	2.6 (1.1–6.5)	3.1 (1.2–6.9)	2.7 (1.1–6.6)	5.6 (2.0–10.1)
Women, %	55.5 (55.4–55.6)	53.2 (52.7–53.7)	55.4 (54.9–56.0)	57.8 (57.2–58.4)	55.5 (55.4–55.6)
Age at study entry, years	48.6 (48.6–48.7)	41.3 (41.2–41.5)	41.3 (41.2–41.5)	42.2 (42.0–42.3)	48.0 (48.0–48.0)
*Social Deprivation*, *%*					
Least Deprived	18.9 (18.9–19.0)	11.3 (11.0–11.6)	4.5 (4.3–4.7)	12.0 (11.6–12.4)	17.7 (17.6–17.8)
Most Deprived	19.7 (19.7–19.8)	29.5 (29.0–29.9)	58.7 (58.1–59.2)	31.0 (30.5–31.6)	21.9 (21.6–22.0)
Consultations in year before study	5.5 (5.5–5.5)	6.2 (6.1–6.2)	5.7 (5.7–5.8)	5.2 (5.1–5.3)	5.5 (5.5–5.6)
*Smoking status*, *%*					
Current smokers	17.3 (17.2–17.4)	17.3 (17.2–17.4)	12.6 (12.3–13.0)	14.3 (13.9–14.8)	17.0 (16.9–17.1)
Ex-smokers	19.8 (19.7–19.9)	9.4 (9.1–9.7)	9.9 (9.5–10.3)	14.9 (14.4–15.4)	18.9 (18.8–19.0)
Never smokers	62.9 (62.8–63.1)	78.0 (77.5–78.4)	75.8 (75.3–76.4)	67.0 (66.3–67.6)	64.1 (64.0–64.2)
Diabetes mellitus, %	2.5 (2.5–2.6)	6.0 (5.8–6.2)	4.6 (4.3–4.8)	2.9 (2.7–3.1)	2.7 (2.7–2.8)
Hypertensive, %	6.0 (6.0–6.1)	4.2 (4.0–4.4)	6.7 (6.5–7.0)	3.8 (3.6–4.1)	5.9 (5.9–6.0)
SBP, mmHg	130.6 (130.5–130.6)	122.9 (122.7–123.1)	127.1 (126.8–127.3)	122.6 (122.3–122.9)	129.9 (129.8–129.9)
DBP, mmHg	78.6 (78.5–78.6)	76.8 (76.7–77.0)	78.6 (78.5–78.8)	76.2 (76.1–76.4)	78.4 (78.4–78.5)
BMI, kg/m2	26.6 (26.6–26.7)	25.6 (25.5–25.7)	27.8 (27.7–27.9)	25.4 (25.3–25.5)	26.6 (26.6–26.6)
Total cholesterol, mmol/L	5.5 (5.5–5.5)	5.1 (5.0–5.1)	5.0 (5.0–5.1)	5.2 (5.2–5.3)	5.4 (5.4–5.4)
HDL cholesterol, mmol/L	1.4 (1.4–1.4)	1.2 (1.2–1.2)	1.4 (1.4–1.4)	1.4 (1.3–1.4)	1.4 (1.4–1.4)
Statin use, %	2.5 (2.4–2.5)	4.6 (4.4–4.8)	3.0 (2.8–3.2)	3.2 (3.0–3.4)	2.6 (2.5–2.6)
Anti-hypertensive drug use, %	15.7 (15.6–15.8)	12.5 (12.1–12.8)	15.1 (14.7–15.4)	10.5 (10.2–10.9)	15.4 (15.4–15.5)
Oral contraceptives/HRT use, %^a^	29.2 (29.1–29.3)	19.7 (19.2–20.3)	21.0 (20.4–21.6)	22.2 (21.6–22.9)	28.4 (28.3–28.6)

Unless indicated otherwise, values given are means (95% confidence intervals); BMI indicates body mass index; DBP, diastolic blood pressure; HDL, high density lipoprotein; SD; standard deviation; SBP, systolic blood pressure; HRT, hormone replacement therapy.

^a^ In women only.

**Fig 1:**
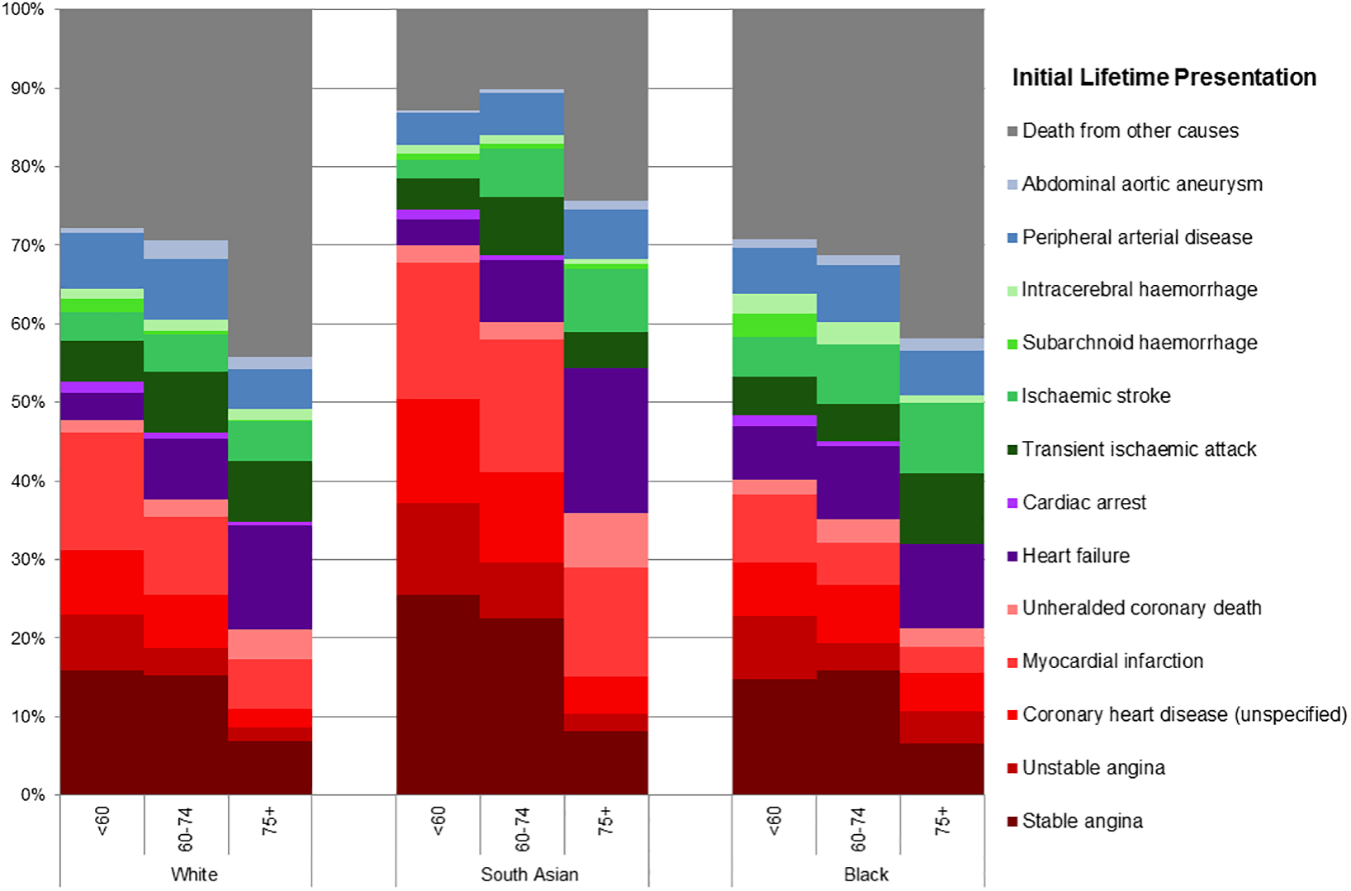
Differences between ethnic groups in coronary, cardiac, cerebrovascular, abdominal and peripheral arterial disease diagnoses as a proportion of total incident cardiovascular disease and deaths from other causes, in three age bands (<60, 60–74, 75+).

We found considerable and significant differences between the three ethnic groups in the proportion of specific CVDs with which cardiovascular disease was first diagnosed (Fig 1). Coronary heart disease (CHD) diagnoses (stable and unstable angina, unspecified coronary heart disease, myocardial infarction and unheralded coronary death) predominated in South Asians, particularly at younger ages, compared to both White and Black patients. 69.9% (67.2%-72.4%) of initial CVD diagnoses were CHD in South Asian patients aged less than 60 years compared to 47.8% (47.3%-48.3%) in White and 40.1% (36.3%-43.9%) in Black patients of the same age. South Asian patients were substantially less likely to die from non-CVD causes before initial lifetime diagnosis with CVD (12.9% (11.2%-14.9%) than either White (27.8% (27.4%-28.2%)) or Black patients (29.3% (25.9%-33.0%)) because of this predominance of coronary disease. Compared to White patients, South Asians and Blacks were about 10 years younger at initial lifetime diagnosis of CVD. Age at initial diagnosis further varied by gender within ethnicity: White women had their initial lifetime diagnosis 13 years later than Black women, while White men had their first diagnosis 8 years after South Asian men(Table 2).

**Table 2:** Age at cardiovascular disease onset in years.

	White	South Asian	Black	Mixed/Other	Total
All patients	71.2 (71.1–71.3)	61.5 (60.9–62.0)	62.1 (61.2–62.9)	67.2 (66.5–67.9)	71.0 (70.9–71.0)
Men	68.2 (68.1–68.3)	60.4 (59.7–61.1)	62.8 (61.7–63.9)	64.1 (63.1–65.1)	68.0 (67.9–68.1)
Women	74.2 (74.1–74.3)	62.9 (62.0–63.8)	61.4 (60.3–62.5)	70.2 (69.2–71.3)	73.9 (73.8–74.0)

**Fig 2:**
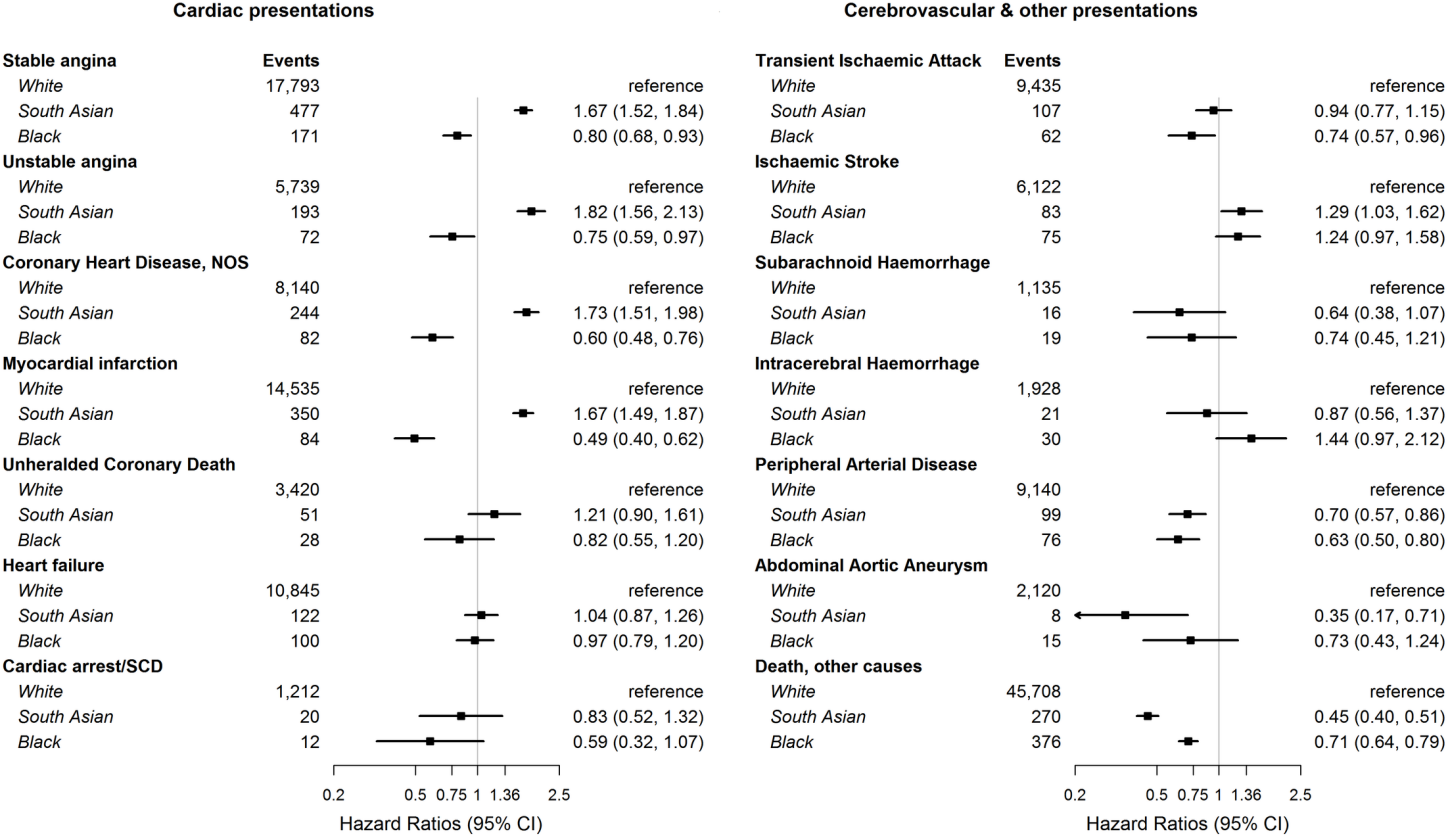
Association between ethnic group and initial lifetime diagnosis of coronary, cardiac, cerebrovascular, abdominal and peripheral arterial diseases and deaths from other causes, adjusted* for age and sex. Hazard ratios (HRs) of South Asian and Black patients compared to White patients; *adjustments included age, quadratic age, sex and stratification by primary care practice.

Compared with White patients, Black patients had significantly lower age-sex adjusted hazard ratios (HRs) for all the coronary disease diagnoses, while South Asian patients had significantly higher HRs (Fig 2). We found no ethnic differences in associations with heart failure presentations. Ethnic associations with stroke diagnoses were more variable: Compared to White patients, we found excess hazards for South Asian patients for ischaemic stroke (HR 1.29 (1.03–1.62)) and excess hazards approaching significance for Black patients for both ischaemic stroke (1.24 (0.97–1.58)) and intracerebral haemorrhage (1.44 (0.97–2.12)). There were no significant ethnic differences in associations with subarachnoid haemorrhage, possibly due to the small number of events. Results for a composite stroke endpoint also approached significance (Black 1.18 (0.98, 1.44); South Asian 1.07 (0.89, 1.30)). South Asian patients had significantly lower HRs for PAD and AAA, while Black patients had lower HRs for PAD only.

**Fig 3:**
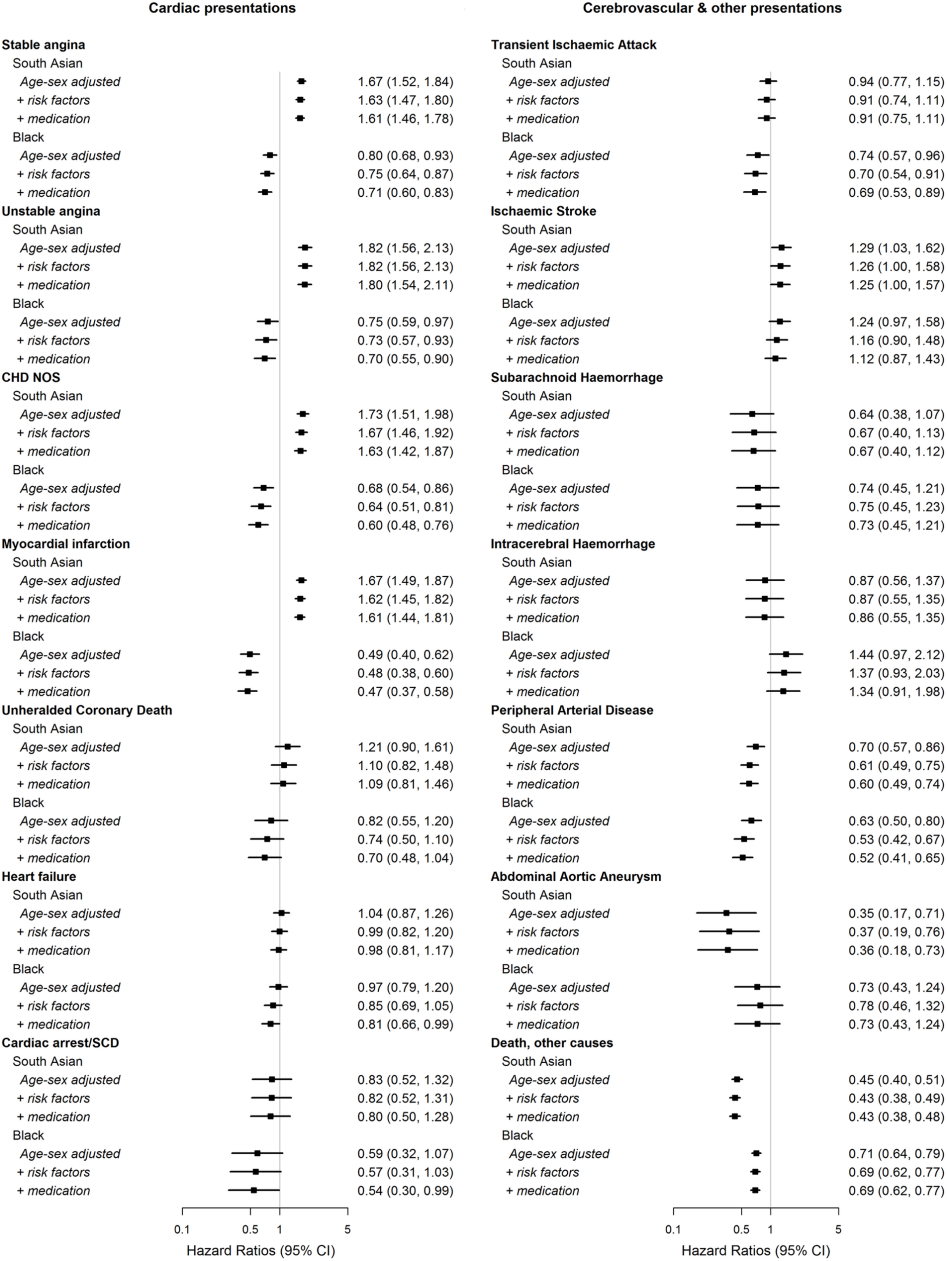
Association between ethnic group and initial lifetime diagnosis of coronary, cardiac, cerebrovascular, abdominal and peripheral arterial diseases and deaths from other causes, adjusted for age and sex*, CVD risk factors**, and medications***. Hazard ratios (HRs) of South Asian and Black patients compared to White patients; *adjustments for age and sex included age, quadratic age, sex and stratification by primary care practice; adjustments for CVD risk factors further included deprivation, smoking, diabetes, systolic blood pressure, body mass index, total cholesterol, and HDL cholesterol; ***adjustment for medications further included statin use, anti-hypertensive drug use and oral contraceptives/HRT use in women only; SCD indicates sudden cardiac death, NOS, not otherwise specified.

Adjustment for cardiovascular risk factors and medications, using multiple imputation to handle missing data, made little difference to the associations between ethnic group and the range of initial diagnoses (Fig 3).

Compared to White patients of the same age, the increased hazard of stable angina and CHD NOS in South Asians aged 30 to 59 was significantly greater, with the excess risk also raised in patients aged 60–74 but not older (S3 Fig). The hazards of the other cardiovascular diagnoses were not significantly modified by age group for both South Asian and Black groups, although the initial diagnosis with heart failure in Black patients compared to White showed a declining hazard with age. The associations between ethnic group and initial diagnosis of CVD were generally not modified by sex except for TIA in Black patients (S4 Fig).

Restricting endpoints to those from secondary care and mortality made no material difference to the associations between ethnic group and specific initial diagnoses of CVD. Similarly there was little difference in the association of ethnic group with all CVDs (except ischaemic stroke) prior to and after 2006 when incentivisation for recording of ethnic group began, despite a marked increase in recording of ethnicity data after this point (S5 Fig). The complete case analysis was underpowered to detect any meaningful association between ethnicity and the full range of cardiovascular disease endpoints (S6 Fig).

## Discussion

In this large population-based cohort of over 1 million patients, with a median of 5.7 observation years and more than 95,000 events, we found strong evidence of heterogeneity in both size and direction of associations between ethnic group and 12 different CVD presentations. We demonstrated overwhelming predominance of CHD diagnoses as the first lifetime expression of CVD in South Asian patients. In Black patients increased hazards of ischaemic and haemorrhagic stroke were consistent with previous studies but we could not rule out a null association.[13,43,44] The associations we found were generally robust to adjustment for cardiovascular risk factors and medication use.

The predominance of CHD diagnoses in South Asians was particularly strong in the youngest age group (less than 60 years), with 70% of all first lifetime diagnoses being different manifestations of coronary disease, compared to 48% in White patients and 40% in Black. South Asian patients were substantially less likely to die than either White or Black patients from a non-CVD cause of death than they were to have CHD diagnosed. The striking finding does suggest that serious consideration should be given to prioritising younger South Asian patients for cardiovascular risk assessment, through programmes such as the NHS Health Checks programme.

Black patients were significantly less likely than White to be diagnosed with one of the coronary heart disease diagnoses as a first CVD diagnosis, which is not consistent with previous UK EHR studies[5,15] or US studies on ethnic differences.[18,44] Critically, however, our findings are consistent with those which investigated CHD within a competing risk framework in both the UK[43] and the US,[24] indicating the importance of taking account of other possible first CVD diagnoses or deaths from other causes in understanding ethnic differences. Differences in access to healthcare, which is free at the point of delivery in the UK but often based on ability to pay in the US, may also play a role in explaining our findings.[45]

Our finding of no association of incident heart failure with ethnic group differs from previous findings both in the UK[14] and the US[22,23], though consistent with a large study of heart failure prevalence with adequate representation of ethnic minority groups.[46] Unlike the US studies, the Black patients in our study had rates of hypertension comparable to White patients. Additionally, our focus on initial lifetime presentation within a competing risk framework of a range of CVDs would exclude cases of heart failure which were sequelae of myocardial ischaemia, unlike other studies of incident heart failure, and may also explain differences to previous studies.

The association with individual stroke types as well as a composite stroke endpoint for Black patients differs in size but not direction found in previous stroke incidence studies.[13,43] The size of association we found for Black patients *was* comparable to a major US study on ethnicity and CVD endpoints which used a competing risk framework. Unlike that study, we did not find an increased risk of deaths from non-CVD causes for Black patients compared to White patients.[12,24,43] Our finding that South Asians had a reduced hazard of PAD compared to White patients is consistent with existing studies,[19] while the reduced hazard in Black patients is not.[19,47] The Black patients in our study were significantly less likely to be current or ex-smokers and had comparable rates of hypertension compared the White patients, which may explain the lower hazard of PAD.[48]

Adjustment for common CVD risk factors, including diabetes, did not change the associations we found between ethnic group and our endpoints, which is consistent with a recent study on ethnic differences for CHD but not stroke or heart failure. [23,49] Previous work on the association of type 2 diabetes and a range of endpoints using CALIBER data did not find differences between ethnic groups.[37]

Our results demonstrate the important contribution cohorts constructed from clinically collected electronic health records can provide in the understanding of relative risk of different diseases between ethnic groups, complementing findings from bespoke investigator-led cohort studies.

### Limitations

While one of the strengths of our study is the size of cohort we were able to construct, one limitation of our study is the large number of patients we excluded because their ethnicity was not recorded (S2 and S3 Tables compare those excluded from the cohort because of unrecorded ethnicity with the cohort patients.). We cannot exclude possible impact on our results of this selection bias, although we note our data sources have been found to be representative of the English population in terms of ethnicity.[33]

Recording of ethnic group in primary care settings increased significantly after incentivisation payments started in 2006, with a differential increase in recording of South Asian and Black ethnic groups. (See S6 Fig) While our sensitivity analysis found no difference in the association of ethnic group with our CVD outcomes pre and post incentivisation, the consequences of this increase was the mean observation time for patients from South Asian and Black groups was approximately half of that for White patients and patients from these groups were more likely to be censored for administrative reasons (transferred out of the practice or end of study). It is possible that if these groups were observed for longer, more events and different associations with CVD diagnoses might have been observed. In order to assess the possible impact of this bias in observation time on our results, we estimated the age-sex adjusted HRs for all end points when we censored patients at the median observation time for the South Asian patients—2.3 years in men and 2.9 years in women—if they had not had an initial diagnosis or been censored for other reasons before then. For this post-hoc sensitivity analysis, we had a total of 27,982 cardiovascular events, 30% of the events in the uncensored cohort. While the HRs were slightly attenuated, the relative differences between the ethnic groups remained unchanged (S7 Fig), which suggests that differences in observation times between the ethnic groups is not a significant source of bias in our results.

We used relatively broad categories for ethnic group, which may mask differences within these categories, such as differences between African and Caribbean patients or Pakistani, Bangladeshi and Indian patients, as has been found in other studies [6,13,14,50]. We note that the HR for our composite South Asian group for incident myocardial infarction is similar to that found in Scotland for Pakistani patients.[15]

Although a strength of this study is the ability to investigate a wide range of cardiovascular diseases with significant number of events in the main ethnic groups, a number of potential risk factors were not recorded in our data sources. We cannot exclude unmeasured confounding due to diet, physical activity, country of birth,[51] experience of racism, ethnic density,[52] other environmental factors, individual measures of socio-economic status,[53], or other unknown factors. We were, however, able to include measures of small area deprivation which has been shown to be associated with coronary heart disease independently of individual socio-economic status.[54] Additionally, we must recognise the possibility of errors in the individual EHR data sources [55,56], which could lead to misattribution of different endpoints. Nonetheless, there is good evidence for the validity of our risk factors and disease endpoints. First, a recent systematic review of studies validating diagnoses in CPRD found a median positive predictive value of 88% across a wide range of diagnoses,(7) while a separate systematic review found the accuracy of discharge coding in HES to be 83%,[56] indicating the general validity of these data sources for identifying clinical disease. Second, using identical definitions for these same 12 diseases in a larger cohort, we have replicated anticipated risk factor/ disease associations with age and gender,[30] systolic and diastolic blood pressure,[27] type 2 diabetes,[37] smoking,[31] socioeconomic deprivation,[29] depression,[57] and alcohol.[58]

### Public health and clinical implications

Our findings suggest that screening for cardiovascular disease should be prioritised in South Asian patients, especially in the under 60s, compared to other ethnic groups. Currently, the NHS Health Checks, a national programme screening for vascular disease in patients without clinical diagnosed disease, does not specifically mandate prioritising South Asians for risk assessment.[59] Additionally, QRISK2,[60] the only cardiovascular screening tool currently recommended for use in the UK by the National Institute for Health and Clinical Excellence,[61] whether for individual patients or to prioritise patients for programmes such as NHS Health Checks,[62] has been found to under-predict the risk of CVD in this very population.[63] QRISK2 also does not include peripheral arterial disease as an endpoint in the risk prediction calculation, which is likely to underestimate CVD risk in White patients, for whom PAD is a significant initial diagnosis.

Given the substantially younger median age of first disease diagnosis in Black and South Asian women compared to White, clinicians should be encouraged to be alert to the possibility of cardiovascular disease in younger women from these minority groups. Further research to understand better the interplay between age, ethnic group and gender is needed to elucidate explanations for this difference.

### Research implications

The heterogeneity of association between ethnicity and different CVDs highlights the importance of considering the full range of cardiovascular disease presentations in studies of ethnicity, as well as the role of alternate presentations play in competing risks for individual CVDs. Better recording of ethnic group in primary care since 2006 will enable further research in this area with longer follow-up time as additional data years becomes available. Inclusion of better area measures of ethnic composition and other measures of small neighbourhood character as potentially mediating factors in the relationship between CVD and ethnicity should be considered. Mixed ethnic groups have been increasing over time, which may mean that ethnic groups in the UK are becoming less distinct with time. In the future it may be clinically useful to think about genotype directly, rather than ethnicity.

We note with interest the number of patients across all ethnic minorities who remain free of clinical diagnoses of CVD to age 75. Further research to investigate ethnic differences in those who manage to avoid these common diseases into older age would add to the literature in this area.[64,65]

## Conclusions

Our study reinforces and amplifies the importance of incident coronary heart disease for South Asians, particularly those under the age of 60, raising the question about whether they should be prioritised for cardiovascular risk assessment in programmes like NHS Health Checks. We reassuringly found no difference between ethnic groups in initial presentation with heart failure and a smaller than anticipated excess relative risk of the stroke presentations in Black patients. We have also identified the importance of considering the full range of cardiovascular disease presentations so that opportunities for secondary prevention are not missed. We also found differences between ethnic groups in the proportions with modifiable cardiovascular risk factors, specifically higher prevalence of diabetes and hypertension in Asian and Black groups which could indicate areas for targeting of prevention education for different ethnic groups.

## Supporting information

S1 Appendix Approach to imputation. (DOCX)

S1 Table Baseline co-variates by ethnic group and age bands. (DOCX)

S2 Table Summary characteristics of patients with recorded and unrecorded ethnicity among eligible patients. (DOCX)

S3 Table Proportion of patients with events in patients with recorded and unrecorded ethnicity among eligible patients. (DOCX)

S1 Fig Study flow diagram. (TIF)

S2 Fig Algorithm defining ethnic group. (TIF)

S3 Fig Sex-adjusted hazard ratios for the association of ethnic group with 12 CVDs by baseline age group. (TIF)

S4 Fig Age-adjusted hazard ratios for the association of ethnic group with 12 CVDs in men and women. (TIF)

S5 Fig Changes in ethnicity coding over time. (TIF)

S6 Fig Hazard ratios for the association of ethnic group with 12 CVDs, adjusted for age and sex, key risk factors, and medication use in complete cases. (TIF)

S7 Fig Age and sex adjusted hazard ratios for the association of ethnic group with 12 CVDs with observation time censored at median observation time for South Asian men and women. (TIF)
