# Supplementary material for: Ethnicity and the first diagnosis of a wide range of cardiovascular diseases: Associations in a linked electronic health record cohort of 1 million patients
Source: PLoS One. 2017 Jun 9;12(6):e0178945. doi: 10.1371/journal.pone.0178945 (PMC5466321; doi:10.1371/journal.pone.0178945)
Supplement: S1 Appendix — (DOCX) [file pone.0178945.s001.docx]

**S1 Appendix – Approach to Imputation**

Baseline smoking status and risk factor data appeared to be missing at random after adjusting for major confounders (e.g. age, sex, diabetes, BMI and blood pressure). Hence, multiple imputation was implemented using the MI command in the statistical package STATA, to replace missing values in exposure and risk factor variables.^1^ Imputation models were estimated separately for men and women and included:

1. all the baseline covariates used in the main analysis (age, quadratic age, BMI, diabetes, smoking, systolic blood pressure, total cholesterol, HDL cholesterol, index of multiple deprivation quintile);
2. prior (between 1 and 4 years before study entry) and post (between 0 and 1 year after study entry) averages of continuous covariates in the main analysis;
3. baseline measurements of covariates not considered in the main analysis (diastolic blood pressure, white cell count, haemoglobin, creatinine, alanine transferase, number of consultations in year);
4. baseline medications (statins, blood pressure lowering medication, low-dose aspirin, diuretics, oral contraceptives and hormone replacement therapy);
5. coexisting medical conditions (diagnosed hypertension, history of depression, cancer, renal disease, liver disease and chronic obstructive pulmonary disease);
6. the Nelson-Aalen hazard and the event status for each of the 12 endpoints analysed;^2^

Non-normally distributed variables were log-transformed for imputation and exponentiated back to their original scale for analysis. Five multiply imputed datasets were generated, and Cox models were fitted to each dataset. Coefficients were combined using Rubin’s rules. We checked whether the imputations were plausible by comparing plots of the distribution of observed and imputed values of all variables.

**References**

1. Van Buuren S. Multiple imputation of discrete and continuous data by fully conditional specification. *Stat Methods Med Res*. 2007;16:219–242.

2. White IR, Royston P. Imputing missing covariate values for the Cox model. *StatMed*. 2009;28:1982–1998.
