# Supplementary material for: Ethnicity and the first diagnosis of a wide range of cardiovascular diseases: Associations in a linked electronic health record cohort of 1 million patients
Source: PLoS One. 2017 Jun 9;12(6):e0178945. doi: 10.1371/journal.pone.0178945 (PMC5466321; doi:10.1371/journal.pone.0178945)
Supplement: S1 Table — (DOCX) [file pone.0178945.s002.docx]

**S1 Table - Summary characteristics by ethnic group and three age groups (30-59, 60-74, 75+)**

|  | **White** | | | **South Asian** | | | **Black** | | |
| --- | --- | --- | --- | --- | --- | --- | --- | --- | --- |
|  | **30-59**  N=729,064 | **60-74**  N=169,797 | **75+**  N=72,422 | **30-59**  N=34,401 | **60-74**  N=3,295 | **75+**  N=596 | **30-59**  N=28,036 | **60-74**  N=2,455 | **75+**  N=405 |
| Observation time, yrs | 6.1 (6.1-6.1) | 7.0 (7.0-7.0) | 5.3 (5.3-5.3) | 4.0 (3.9-4.0) | 5.0 (4.9-5.2) | 3.9 (3.6-4.2) | 4.2 (4.2-4.3) | 5.3 (5.1-5.4) | 3.9 (3.5-4.2) |
| Women, % | 54.3 (54.2-54.4) | 55.4 (55.2-55.7) | 67.6 (67.3-67.9) | 52.9 (52.4-53.4) | 55.5 (53.8-57.2) | 58.2 (54.2-62.1) | 55.4 (54.8-56.0) | 56.3 (54.4-58.3) | 53.6 (48.7-58.4) |
| Age, yrs | 41.2 (41.2-41.2) | 66.5 (66.5-66.5) | 81.3 (81.3-81.4) | 38.3 (38.2-38.4) | 66.2 (66.1-66.4) | 79.8 (79.4-80.1) | 38.6 (38.5-38.7) | 66.2 (66.1-66.4) | 79.6 (79.2-80.1) |
| Deprivation |  |  |  |  |  |  |  |  |  |
| Least quintile | 18.5 (18.4-18.6) | 19.1 (18.9-19.3) | 17.5 (17.2-17.8) | 11.2 (10.9-11.5) | 10.5 (9.5-11.6) | 9.9 (07.8-12.6) | 4.4 (04.1-04.6) | 4.5 (03.7-05.4) | 5.0 (03.2-07.6) |
| Most quintile | 20.8 (20.7-20.9) | 17.6 (17.4-17.7) | 19.6 (19.3-19.9) | 30.5 (30.0-31.0) | 28.7 (27.2-30.3) | 31.5 (27.9-35.3) | 59.6 (59.0-60.2) | 57.8 (55.8-59.7) | 51.7 (46.9-56.6) |
| Consultations | 5.1 (5.1-5.1) | 6.3 (6.3-6.3) | 7.7 (7.6-7.8) | 5.9 (5.8-5.9) | 8.8 (8.5-9.0) | 9.2 (8.6-9.8) | 5.5 (5.4-5.5) | 7.8 (7.6-8.1) | 9.3 (8.5-10.0) |
| Smoking status |  |  |  |  |  |  |  |  |  |
| Current | 20.5 (20.4-20.6) | 8.8 (8.7-9.0) | 4.3 (4.1-4.5) | 13.3 (12.9-13.7) | 6.6 (5.7-7.6) | 5.9 (4.1-8.4) | 15.0 (14.6-15.5) | 7.0 (05.9-08.3) | 6.5 (4.3-9.9) |
| Ex- | 18.6 (18.5-18.7) | 24.1 (23.8-24.3) | 21.7 (21.3-22.0) | 9.1 (08.8-09.5) | 11.1 (10.0-12.4) | 14.6 (11.7-18.1) | 9.4 (09.0-09.8) | 14.2 (12.7-15.9) | 19.3 (15.2-24.1) |
| Never | 60.9 (60.8-61.0) | 67.1 (66.8-67.4) | 74.1 (73.7-74.4) | 77.5 (77.1-78.0) | 82.2 (80.7-83.7) | 79.5 (75.6-82.9) | 75.6 (75.0-76.2) | 78.7 (76.8-80.6) | 74.2 (69.0-78.8) |
| Diabetes mellitus, % | 1.6 (1.6-1.6) | 5.2 (5.1-5.3) | 5.7 (5.6-5.9) | 4.3 (4.1-4.5) | 21.4 (20.0-22.8) | 19.1 (16.2-22.5) | 3.0 (2.8-3.2) | 19.3 (17.8-20.9) | 22.2 (18.4-26.5) |
| Hypertensive, % | 4.3 (04.3-04.4) | 11.1 (10.9-11.2) | 11.2 (11.0-11.4) | 3.4 (03.2-03.6) | 10.9 (09.9-12.0) | 8.9 (06.9-11.5) | 5.7 (05.4-06.0) | 17.9 (16.5-19.5) | 11.1 (08.4-14.6) |
| SBP, mmHg | 125 (125-125) | 143 (143-143) | 148 (148-148) | 121 (120-121) | 139 (138-139) | 142 (140-144) | 125 (125-125) | 144 (143-145) | 143 (141-145) |
| DBP, mmHg | 78 (78-78) | 82 (82-82) | 80 (80-80) | 76 (76-77) | 80 (79-80) | 77 (77-78) | 78 (78-78) | 83 (83-84) | 79 (78-80) |
| BMI, kg/m^2^ | 26.6 (26.6-26.6) | 27.3 (27.3-27.3) | 25.4 (25.3-25.5) | 25.5 (25.4-25.6) | 26.6 (26.4-26.8) | 24.7 (24.2-25.3) | 27.8 (27.7-27.9) | 28.7 (28.4-29.0) | 26.9 (26.2-27.6) |
| Total chol, mmol/L | 5.4 (5.4-5.4) | 5.6 (5.6-5.6) | 5.4 (5.4-5.4) | 5.1 (5.0-5.1) | 5.1 (5.0-5.1) | 4.9 (4.8-5.1) | 5.0 (5.0-5.0) | 5.2 (5.1-5.3) | 5.3 (5.1-5.5) |
| HDL, mmol/L | 1.4 (1.4-1.4) | 1.5 (1.5-1.5) | 1.5 (1.5-1.6) | 1.2 (1.2-1.2) | 1.3 (1.3-1.3) | 1.3 (1.3-1.4) | 1.4 (1.4-1.4) | 1.5 (1.4-1.5) | 1.5 (1.4-1.6) |
| Statin use, % | 1.4 (1.3-1.4) | 6.4 (6.2-6.5) | 4.3 (4.1-4.4) | 3.1 (2.9-3.3) | 17.8 (16.6-19.2) | 16.1 (13.4-19.3) | 1.8 (1.6-1.9) | 14.5 (13.2-16.0) | 17.0 (13.7-21.0) |
| BP lowering med., % | 10.4 (10.3-10.5) | 28.6 (28.3-28.8) | 38.9 (38.6-39.3) | 9.6 (9.3-9.9) | 36.1 (34.4-37.7) | 46.5 (42.5-50.5) | 11.6 (11.3-12.0) | 48.0 (46.0-50.0) | 52.8 (48.0-57.7) |
| Female hormones, %^a^ | 36.4 (36.2-36.5) | 13.3 (13.1-13.5) | 1.5 (1.4-1.6) | 21.5 (20.9-22.1) | 6.1 (5.1-7.3) | 0.9 (0.3-2.6) | 22.7 (22.1-23.4) | 4.8 (3.8-6.1) | 1.8 (0.7-4.8) |

Note: Unless indicated otherwise, values given are means (95% confidence intervals); Yrs indicates years; SBP, systolic blood pressure; mmHg, millimetres of mercury; DBP, diastolic blood pressure; BMI, body mass index; kg, kilogram; m, metre; chol., cholesterol; mmol/L, micromole per litre; HDL, high density lipoprotein; med., medication.

^a^ in women only
