# Supplementary material for: Ethnicity and the first diagnosis of a wide range of cardiovascular diseases: Associations in a linked electronic health record cohort of 1 million patients
Source: PLoS One. 2017 Jun 9;12(6):e0178945. doi: 10.1371/journal.pone.0178945 (PMC5466321; doi:10.1371/journal.pone.0178945)
Supplement: S2 Table — (DOCX) [file pone.0178945.s003.docx]

**S2 Table. Summary characteristics of patients with recorded ethnicity (main analysis dataset) and unrecorded ethnicity among eligible patients**

|  | **Ethnicity Recorded** | **Ethnicity Unrecorded** |
| --- | --- | --- |
| N patients | 1,068,318 | 825,685 |
| Women, % | 55.5 (55.4-55.9) | 44.2 (44.1-44.3) |
| Median observation time, years (IQR) | 5.6 (2.0-10.1) | 5.7(2.3-10.1) |
| Mean age at entry, years | 48.0 (48.0-48.0) | 45.2 (45.2-45.2) |
| Mean age at onset (sd), years | 70.9 (70.9-71.0) | 73.5 (73.4-73.6) |
| Number of consultations in year before study entry | 3.4 (3.4-3.4) | 5.5 (5.5-5.6) |
| *Social Deprivation* |  |  |
| Least Deprived | 18.1 (18.0-18.1) | 23.5 (23.4-23.6) |
| Most Deprived | 21.5 (21.4-21.6) | 17.1 (17.0-17.2) |
| *Smoking status* |  |  |
| Current smokers | 17.0 (16.9-17.1) | 14.0 (13.9-14.1) |
| Ex-smokers | 18.9 (18.8-19.0) | 15.0 (14.9-15.1) |
| Never smokers | 64.1 (64.0-64.2) | 70.9 (70.8-71.1) |
| *Medical History* |  |  |
| Diabetes mellitus, | 2.7 (2.7-2.8) | 2.7 (2.7-2.8) |
| *Measurements* |  |  |
| BMI, kg/m^2^ | 26.6 (26.6-26.6) | 26.1(26.1-26.1) |
| Total cholesterol, mmol/L | 5.4 (5.4-5.4) | 5.5 (5.5-5.5) |
| HDL cholesterol, mmol/L | 1.4 (1.4-1.4) | 1.4 (1.4-1.4) |
| SBP, mmHg | 129.9 (129.8-129.9) | 129.0 (128.9-129.1) |
| DBP, mmHg | 78.4 (78.4-78.5) | 78.3 (78.3-78.4) |
| Hypertensive, | 5.9 (5.9-6.0) | 4.1 (4.1-4.2) |
| *Medication Use* |  |  |
| Statins | 2.6 (2.5-2.6) | 1.0 (1.0-1.1) |
| Anti-hypertensive medications | 8.4 (8.4-8.5) | 5.0 (4.9-5.0) |
| Oral contraceptives /HRT^a^ | 28.4 (28.3-28.6) | 24.5 (24.4-24.6) |

Continuous variables are summarized as median (inter-quartile range), except for systolic and diastolic blood pressure, summarized as mean (95 confidence interval). Categorical variables are summarized as percentage (95 confidence interval). Abbreviations: BMI indicates body mass index; DBP, diastolic blood pressure; HDL, high density lipoprotein; HRT, hormone replacement therapy; SBP, systolic blood pressure.

^a^ In women only (N= 957,387).
