# Supplementary material for: Ethnicity and the first diagnosis of a wide range of cardiovascular diseases: Associations in a linked electronic health record cohort of 1 million patients
Source: PLoS One. 2017 Jun 9;12(6):e0178945. doi: 10.1371/journal.pone.0178945 (PMC5466321; doi:10.1371/journal.pone.0178945)
Supplement: S3 Table — (DOCX) [file pone.0178945.s004.docx]

**S3 Table. Number and proportion of patients with events in patients with recorded ethnicity (main analysis dataset) and unrecorded ethnicity among eligible patients**

|  | **Ethnicity Recorded** | **Ethnicity Unrecorded** |
| --- | --- | --- |
| Stable Angina | 18,650 (13) | 4,949 (8) |
| Unstable angina | 6,109 (5) | 1,007 (2) |
| Coronary Heart Disease, NOS | 8,589 (7) | 1,984 (3) |
| Myocardial Infarction | 15,145 (14) | 3,426 (6) |
| Unheralded coronary death | 3,548 (3) | 5,944 (10) |
| Heart failure | 11,173 (7) | 4,261 (7) |
| Cardiac arrest/Sudden Cardiac Death | 1,265 (1) | 531 (1) |
| Transient ischaemic attack | 9,678 (6) | 2,966 (5) |
| Ischaemic stroke | 6,323 (4) | 1,467 (2) |
| Subarachnoid haemorrhage | 1,184 (1) | 489 (1) |
| Intracerebral haemorrhage | 2,001 (1) | 777 (1) |
| Peripheral arterial disease | 9,404 (7) | 2,613 (4) |
| Abdominal aortic aneurysm | 2,155 (2) | 968 (2) |
| All cardiovascular disease | 95,224 (67) | 31,382 (53) |
| Deaths from other causes | 46,913 (33) | 27,575 (47) |
