# Supplementary figures and images for: Ethnicity and the first diagnosis of a wide range of cardiovascular diseases: Associations in a linked electronic health record cohort of 1 million patients
Source: PLoS One. 2017 Jun 9;12(6):e0178945. doi: 10.1371/journal.pone.0178945 (PMC5466321; doi:10.1371/journal.pone.0178945)

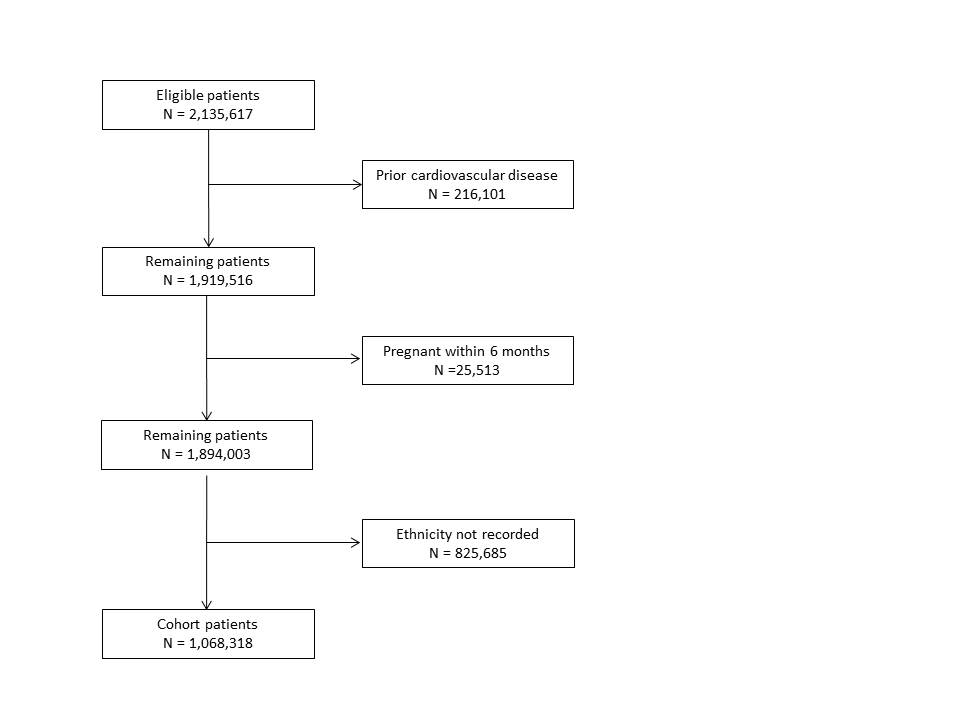

Supplement: S1 Fig — (TIF) [file pone.0178945.s005.tif]

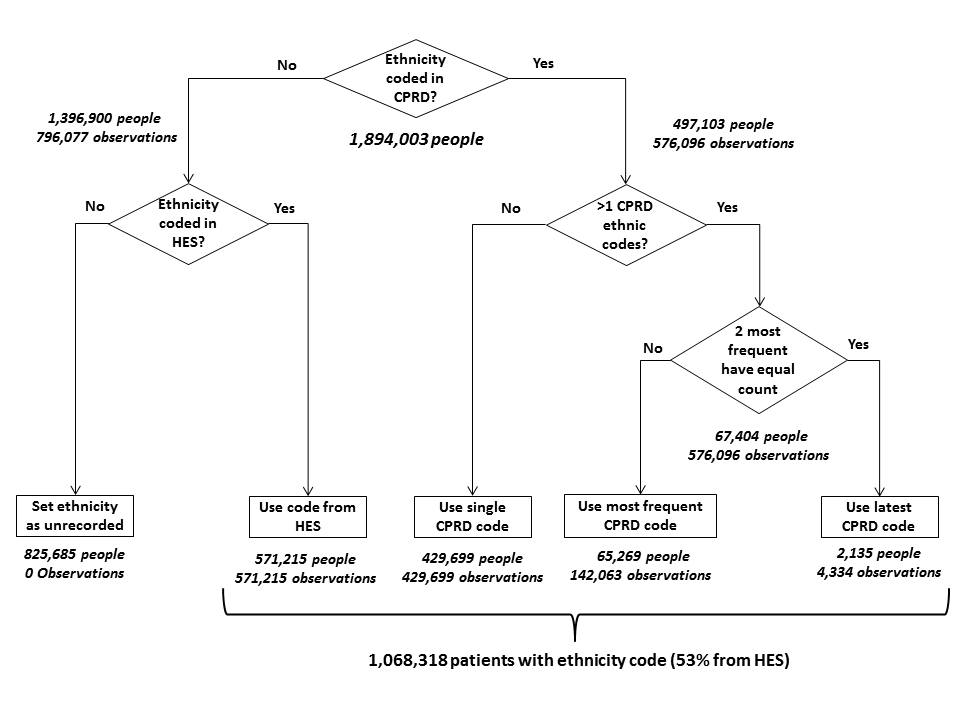

Supplement: S2 Fig — (TIF) [file pone.0178945.s006.tif]

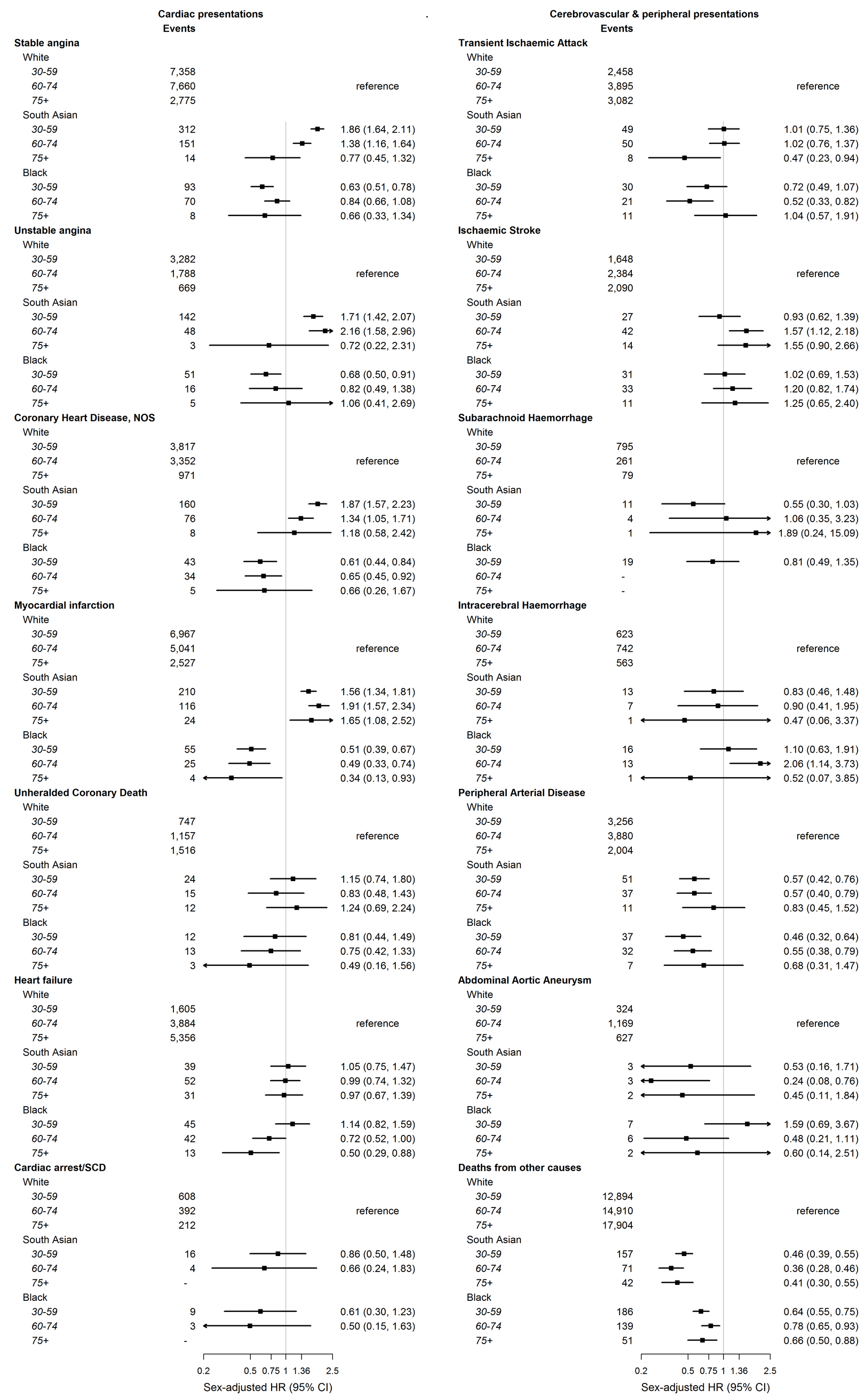

Supplement: S3 Fig — (TIF) [file pone.0178945.s007.tif]

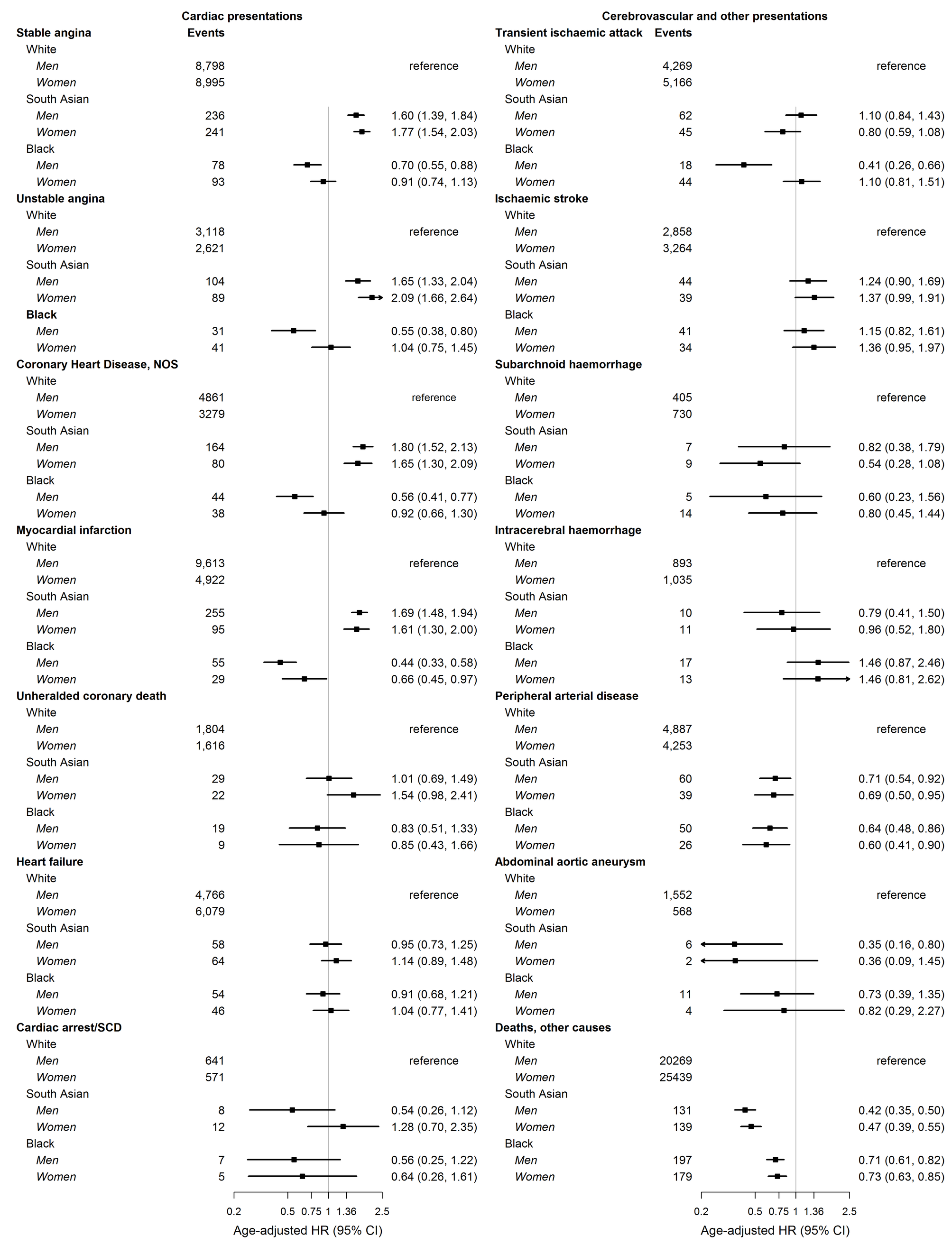

Supplement: S4 Fig — (TIF) [file pone.0178945.s008.tif]

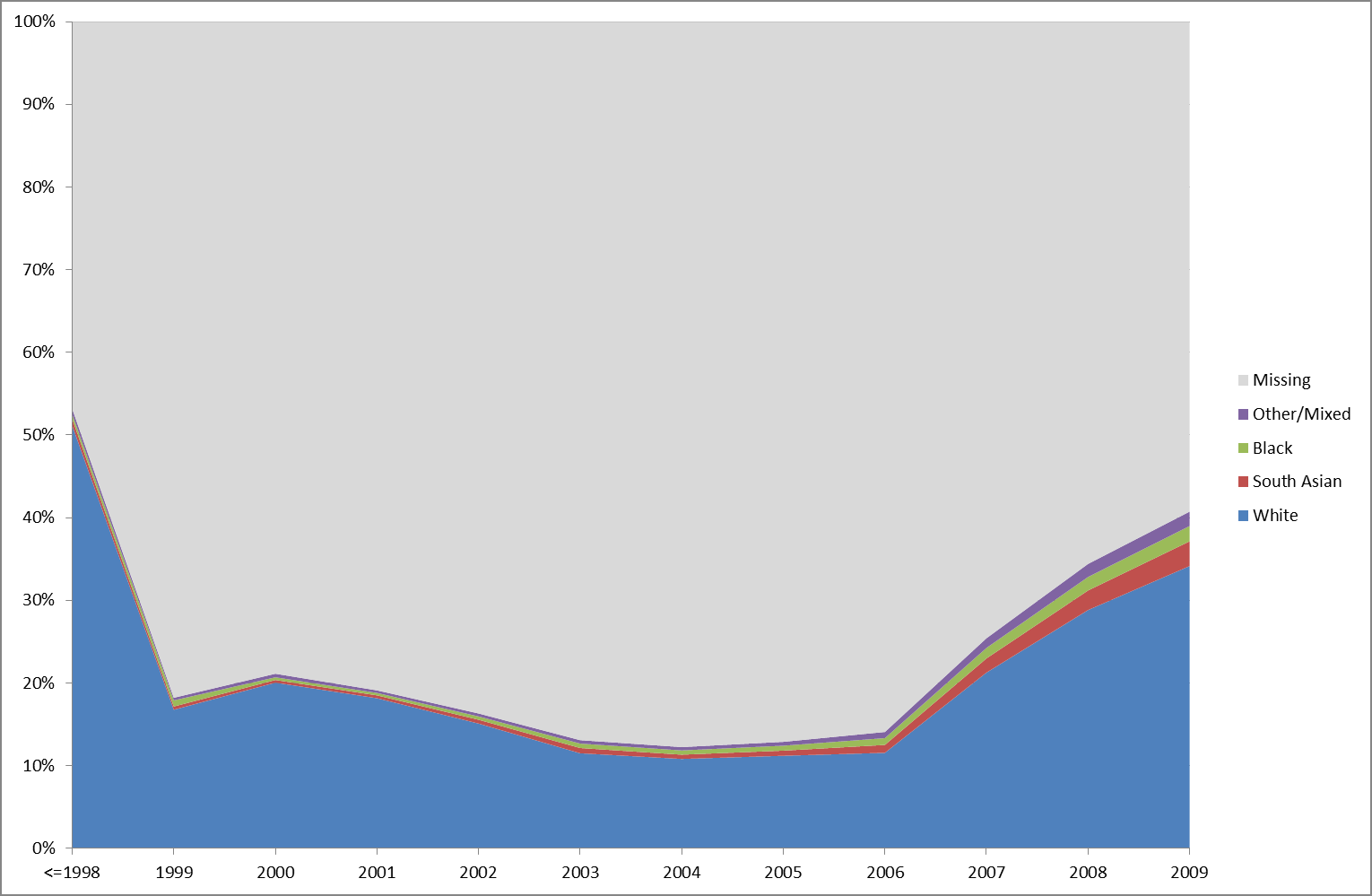

Supplement: S5 Fig — (TIF) [file pone.0178945.s009.tif]

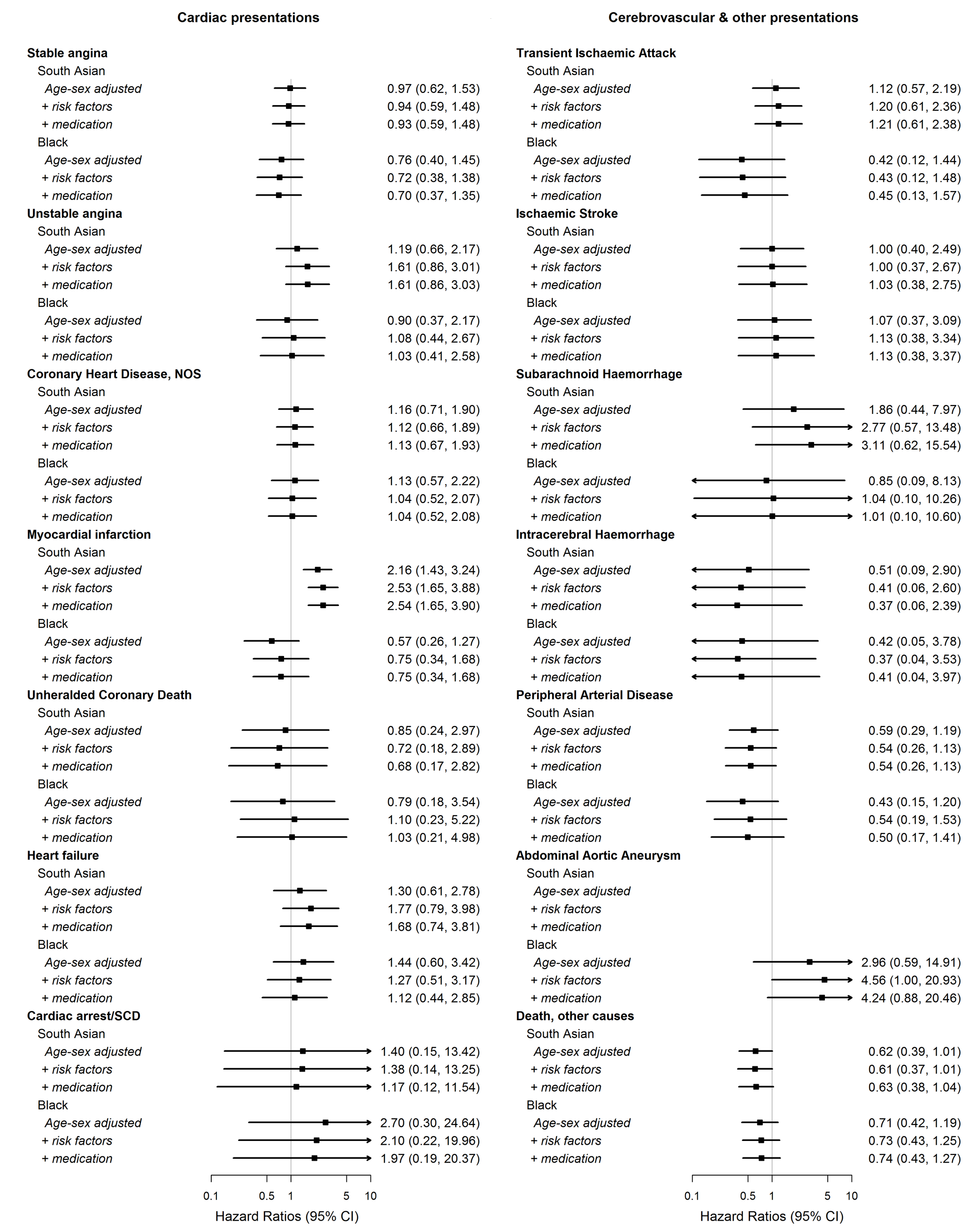

Supplement: S6 Fig — (TIF) [file pone.0178945.s010.tif]

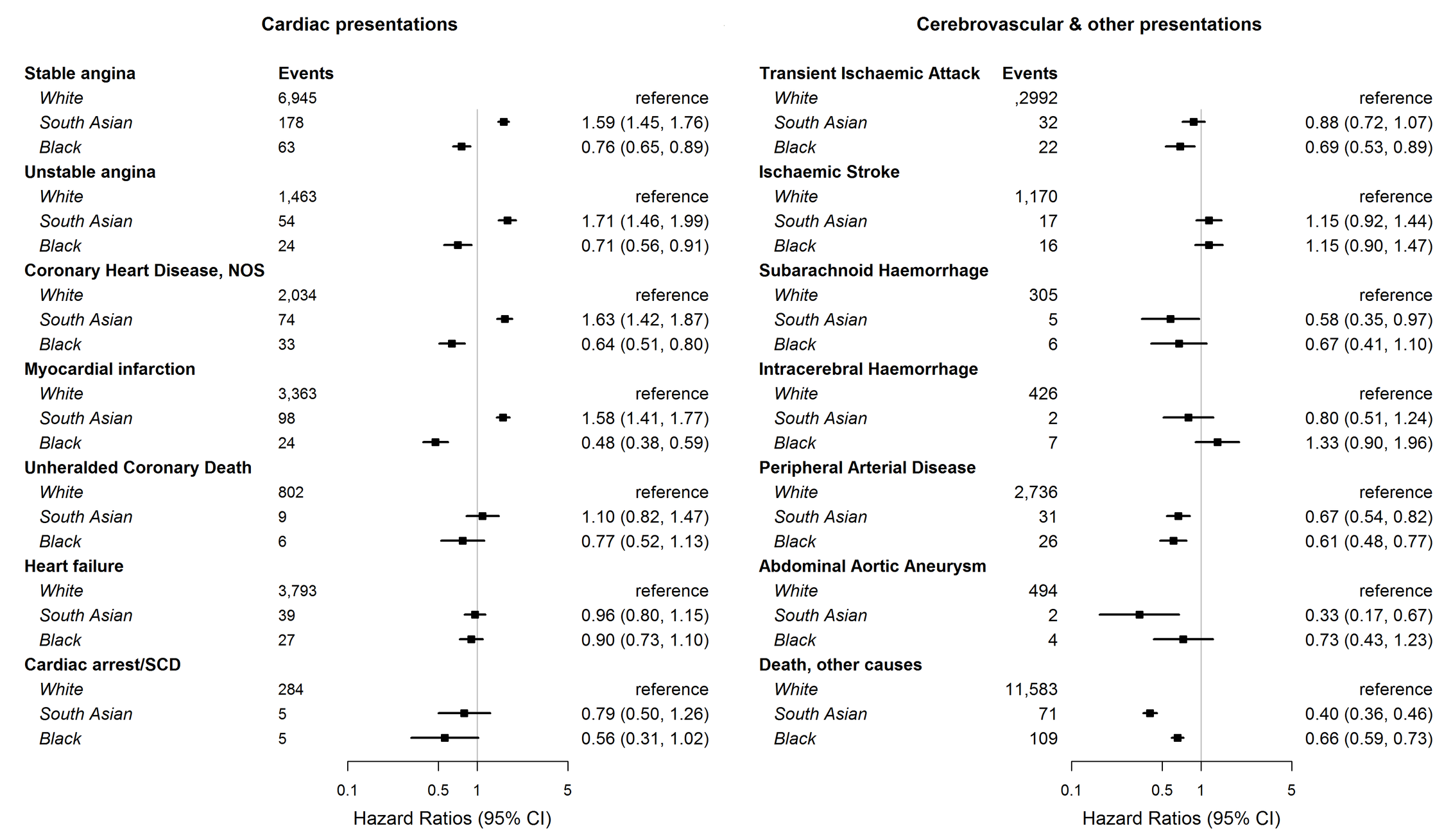

Supplement: S7 Fig — (TIF) [file pone.0178945.s011.tif]
